# A Spatially Resolved View on the Aging Substantia nigra: An Exploratory Proteomic Study

**DOI:** 10.1002/adbi.202500358

**Published:** 2025-09-18

**Authors:** Britta Eggers, Maximilian Hausherr, Michel Lim, Karin Schork, Bilhan Karacora, Robin Grugel, Martin Eisenacher, Isabel Gil Aldea, Peter Riederer, Manfred Gerlach, Katrin Marcus

**Affiliations:** ^1^ Medizinisches Proteom‐Center Medical Faculty Ruhr University Bochum 44801 Bochum Germany; ^2^ Medical Proteome Analysis, Center for Proteindiagnostics (PRODI) Ruhr University Bochum 44801 Bochum Germany; ^3^ Core Unit for Bioinformatics (CUBiMed.RUB), Medical Faculty Ruhr University Bochum 44801 Bochum Germany; ^4^ Navarrabiomed Biobank, Hospital Universitario de Navarra Pamplona 31008 Navarra Spain; ^5^ University Hospital Wuerzburg, Center of Mental Health, Clinic and Policlinic for Psychiatry Psychosomatics and Psychotherapy Margarete‐Hoeppel‐Platz 1 D‐97080 Wuerzburg Germany; ^6^ Psychiatry Department of Clinical Research University of Southern Denmark Odense University Hospital Odense C 5000 Denmark; ^7^ Center of Mental Health Department of Child and Adolescent Psychiatry, Psychosomatics and Psychotherapy, University Hospital of Wuerzburg University of Wuerzburg 97080 Wuerzburg Germany

**Keywords:** brain aging, dementia with Lewy bodies, neuromelanin granules, Parkinson's disease, proteomics

## Abstract

Physiological aging is accompanied by structural and molecular changes in the brain, with varying degrees in different brain areas, and is considered one of the major risk factors for neurodegenerative diseases. Thus, the present study focuses on elucidating age‐related changes in the substantia nigra pars compacta (SNpc), a brain region particularly vulnerable in Parkinson's disease. Here, the aim is to gain a spatially resolved view of aging‐dependent alterations to conclude early processes potentially involved in neurodegeneration. Neuromelanin granules and SNpc tissue are isolated from tissue samples of young and elderly individuals via laser microdissection and measured by mass spectrometry to ascertain changes in protein expression in response to age. The findings include the identification of reduced levels of proteins involved in dopaminergic neurotransmission, either suggesting a specific loss of dopaminergic neurons or a reduction in metabolic activity. Furthermore, increased neuroinflammation is observed in elderly individuals and alterations in vesicular trafficking as well as mitochondrial proteins. Consequently, this exploratory study suggests that alterations causing known pathomechanisms of Parkinson's disease are already occurring in the physiological aging process. Since aging is still the most important risk factor for neurodegenerative diseases, these findings strengthen the necessity for studying age‐related changes.

## Graphical abstract

Although aging is the most important risk factor for several neurodegenerative diseases, the molecular effects of physiological aging are still understudied. By applying spatially‐resolved proteomic analyses of the human substantia nigra pars compacta, alterations in vesicular trafficking and mitochondrial proteins are observed, as well as reduced levels of dopaminergic marker proteins and increased neuroinflammation. Created in BioRender. Wulf, M. (2025) https://BioRender.com/3oj1vmw.

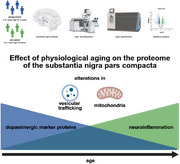

## 1 Introduction

Aging is a multifaceted process that has been identified as a significant risk factor for the development of various diseases. Within the field of neurodegenerative diseases, research has focused on the aging brain due to its sensitivity to the aging process. This process leads to structural changes in brain morphology, size, vasculature and neural interactions resulting in deficits in cognition and an increased risk of stroke and ischaemia.^[^ ^1^, ^2^, ^3^ ^]^ Morphological alterations aberrant in the aging brain of healthy individuals are also found and defined as classical hallmarks of neurodegeneration, such as neuronal loss, gliosis and loss of myelination. At the molecular level, physiological aging as well as early stages of neurodegenerative diseases are often accompanied by increased mitophagy, cellular senescence, a disrupted calcium homeostasis, and an increase in oxidative stress, eventually leading to neuroinflammation.^[^ ^4^, ^5^ ^]^ Furthermore, mitochondrial alterations, protein aggregation, and a disrupted protein clearance system could be identified in both physiological aging and neurodegeneration.^[^ ^6^ ^]^

However, neurodegenerative diseases frequently present with unique pathologies, resulting from a selective vulnerability of specific brain regions and brain cell types.^[^ ^7^ ^]^ This prompts the question of whether changes observed in physiological aging can predict the earliest signs of neurodegeneration. In our recently published study, we sought to elucidate region‐specific alterations in physiological brain aging with a particular focus on the substantia nigra pars compacta (SNpc), a region recognized for its selective vulnerability in Parkinson's disease (PD) and related conditions such as dementia with Lewy bodies (DLB).^[^ ^8^ ^]^ The SNpc presents as a unique part of the brain, being mainly composed of dopaminergic neurons characterized by long, highly branched axons, autonomous activity, and the production of dopamine, a highly reactive neurotransmitter susceptible to oxidation, potentially causing increased oxidative stress.^[^ ^9^ ^]^ Lastly, one key feature of the SNpc is the accumulation of neuromelanin in so‐called neuromelanin granules (NMGs) within the somata of dopaminergic neurons.^[^ ^10^, ^11^, ^12^ ^]^

These NMGs are known to store the pigment neuromelanin (NM) as well as a variety of proteins, lipids, and metals, in particular iron.^[^ ^13^ ^]^ Iron is known to be a major component of oxidative reactions, and NM was identified being the major intraneuronal chelator of iron,^[^ ^10^ ^]^ interestingly, both undergo an age‐dependent increase^[^ ^12^, ^14^ ^]^ and therefore may be directly linked to the unique physiological aging process observed within the SNpc. In addition, NMG‐containing neurons in the SNpc are especially vulnerable in PD and DLB. The correlation between neurodegeneration and physiological aging renders NMGs and the aging SNpc optimal research objects, facilitating the acquisition of insights into aging‐related molecular changes that may elevate the risk of developing neurodegenerative diseases.

So far, numerous studies have been conducted to elucidate age‐specific changes in the human SNpc of individuals without neurological or psychiatric disorders. All studies conclusively identified an enhanced abundance of activated microglia in close proximity to NMGs.^[^ ^4^, ^15^, ^16^ ^]^ This has been shown to be connected to heightened neuroinflammatory signals in elderly subjects, suggesting a strong relation between (healthy) aging and the onset of pathomechanisms that have been previously reported to play a role in PD.^[^ ^4^, ^15^, ^16^ ^]^ In addition, cell culture‐based studies have corroborated these observations and verified NM to be a specific activator of microglia in the human brain, potentially contributing to dopaminergic neuron death.^[^ ^11^, ^17^ ^]^ A recent study performed in a genetically‐modified rodent model, in which brownish pigments, similar to NM are induced through expression of human tyrosinase, an enzyme essential for melanin production in the skin, has additionally demonstrated, that there appears to be a threshold, from which on NM becomes neurotoxic to the dopaminergic neurons, underscoring the clinical relevance of this pigment.^[^ ^18^, ^19^, ^20^ ^]^

In a previous study, utilizing laser microdissection and mass spectrometry to specifically excise tissue of the human SNpc of young, middle‐aged, and elderly subjects, we were able to conclude that the aging SNpc is characterized by accumulation of reactive oxygen species (ROS), increased inflammation, and cytoskeletal remodelling.^[^ ^21^ ^]^

The present studies provide unequivocal evidence that the distinctive composition of the SNpc, characterized by its high density of dopaminergic neurons containing NMGs, exhibits specific age‐related changes. Consequently, when investigating factors of brain aging and their potential association with neurodegeneration, it is imperative to obtain a spatially resolved perspective on age‐related changes. With our previously revised and applied workflow for the precise excision of NMGs and surrounding SNpc tissue deprived of NMGs (SN_Surr._) out of post‐mortem human brain tissue,^[^ ^22^, ^23^, ^24^ ^]^ we are one of the first to provide a spatially resolved picture of the aging SNpc and thereby determine SNpc‐specific hallmarks of aging, potentially revealing unique features leading to the development of PD. Using SN_Surr._ tissue and NMGs of young (mean age 27.8 years) and old individuals (mean age 76 years), we determined protein levels of dopaminergic and glial cells, identified an increase of proteins associated with neuroinflammation in elderly individuals, alterations in the clathrin‐mediated endocytosis, mitochondrial dysfunction, and changes in the abundance of stress granule‐associated proteins. With this, we showcase that early signs of neurodegeneration are indeed developing during the healthy aging process.

## 2 Results

### 2.1 The Aging SNpc is Characterized by a Decreased Dopaminergic Phenotype

**Figure 1:**
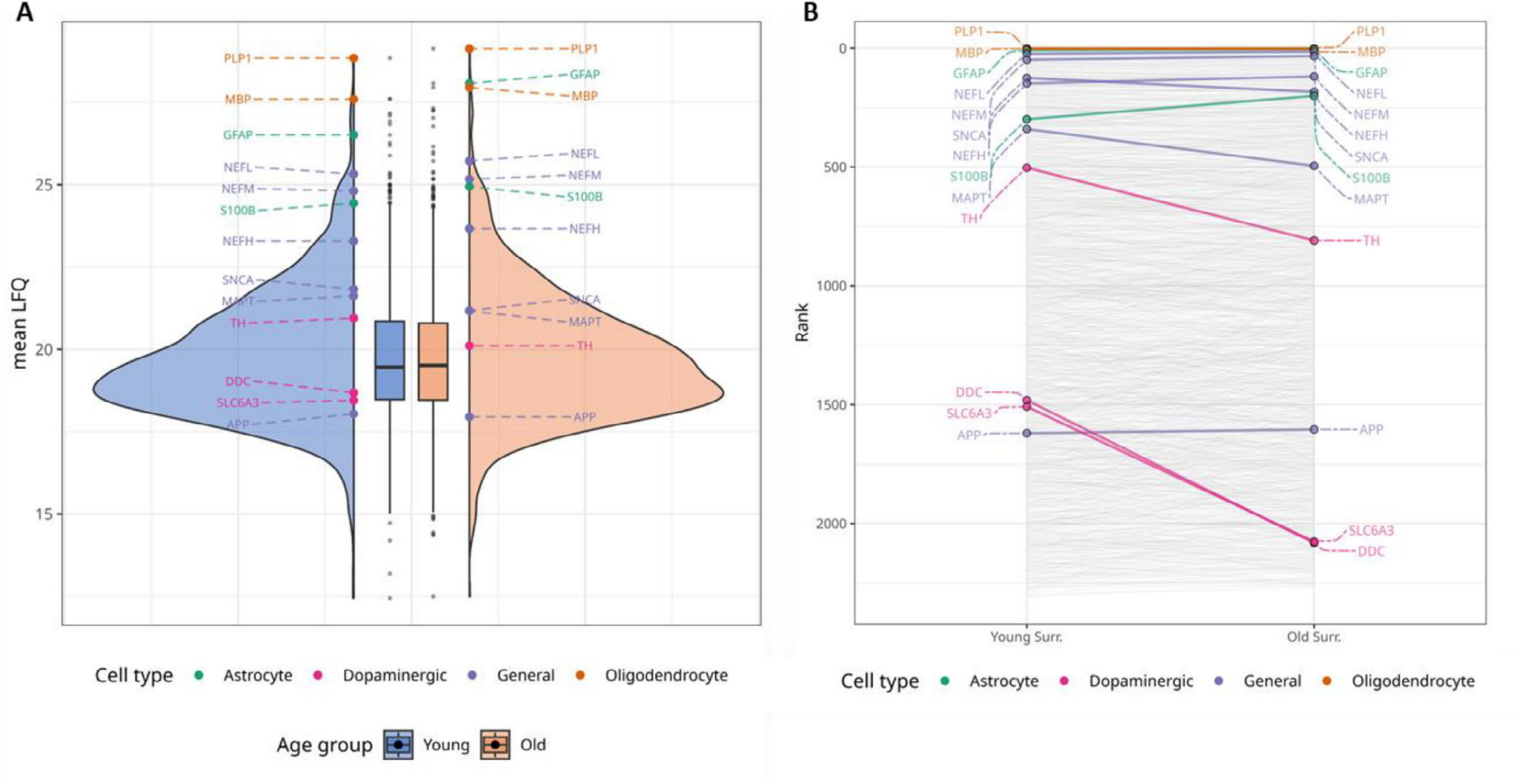
Dopaminergic and astrocytic proteins show abundance changes between both age groups. For each major cell type, marker proteins were selected and screened for their abundances based on label‐free quantified (LFQ) values (A) and normalized iBAQ values (B) in young SN_Surr._ tissue (*n* = 4, mean age: 27.8 years) and old SN_Surr._ tissue (*n* = 4, mean age 76 years). Representative trends of mean values are shown in the graphs. iBAQ values were normalized and averaged for each group. Quantified proteins were ranked by descending iBAQ values and thus overall abundance within the SN_Surr._ Proteins, being indicative of different brain cell types and their position within the dynamic proteome, are highlighted. For extended data, see Supplementary Material S1 (Supporting Information). While oligodendrocytic (orange, Myelin basic protein (MBP), Myelin proteolipid protein (PLP1)) and general neuronal markers (purple, Neurofilament light polypeptide (NEFL), Neurofilament medium polypeptide (NEFM), ß‐Amyloid‐precursor protein (APP), alpha‐Synuclein (SNCA), Microtubule‐associated protein tau (MAPT)) remain unaffected by aging, astrocytic proteins (green, Glial fibrillary acidic protein (GFAP), Protein S100‐B (S100B)) are higher abundant in samples of old tissue donors, while a decreased abundance becomes observable for markers of dopaminergic neurons (pink, (Tyrosine 3‐monooxygenase (TH), Dopa‐Decarboxylase (DDC), Sodium‐dependent dopamine transporter (SLC6A3)). The quantification of DDC and SLC6A3 in aged SN tissue was solely possible on the level of iBAQ values.

To study spatially‐resolved age‐related changes within the SNpc, NMGs were specifically isolated from SNpc brain tissue of young (mean age: 27.8 years) and old individuals (mean age: 76 years). Additionally, SNpc tissue, now devoid of NMGs (SN_Surr._), was isolated to gain a comprehensive understanding of age‐related changes that occur in the SN_Surr._ samples in our young and old cohorts. The SNpc is composed of a variety of cell types, including macroglia like astrocytes, oligodendrocytes, and microglia, as well as non‐dopaminergic and dopaminergic neurons. This diversity of cell types makes the SNpc a representative sample of common age‐related changes that occur in this brain region. In order to ascertain any potential shifts in tissue composition consequent to increasing age, the abundance of specific marker proteins for each major cell type of the SNpc was examined on the level of label‐free quantified (LFQ) intensities and intensity‐based absolute quantification (iBAQ) values (see **Figure** **1**; Supplementary Material S1, Supporting Information). We chose to examine said markers on both levels, since LFQ intensities are normalized between samples on the basis of peptide retention times and peak alignments. iBAQ values instead are calculated on the basis of non‐normalized intensities by dividing the sum of all peptide intensities assigned to one protein in one sample by the number of theoretical peptides. Therefore, it can occur that a protein has no LFQ value but an iBAQ value.

As expected, both values led to similar results concerning the abundance of cell type markers. Several general neuronal markers remained unaffected by age, whereby alpha‐Synuclein (SNCA) and Microtubule‐associated protein tau (MAPT) showed a slight age‐related downshift. Of interest, a pronounced downward shift in dopaminergic neuron markers, among them Tyrosine 3‐monooxygenase (TH), Dopa‐Decarboxylase (DDC) and Sodium‐dependent dopamine transporter (SLC6A3) was visible in the old cohort. However, the quantification of DDC and SLC6A3 in aged SN_Surr._ tissue was solely possible on the level of iBAQ values, indicating a pronounced loss of these proteins in aged SN tissue. The two astrocytic markers, Glial Fibrillary Acidic Protein (GFAP) and Protein S100‐B (S100B), instead displayed higher expression profiles in the old cohort. The oligodendrocytic markers Myelin basic protein (MBP) and Myelin proteolipid protein (PLP1) remained unaffected by age.

### 2.2 The Aging SNpc is Characterized by Increased Neuroinflammation and an Altered Vesicle Transport Machinery

**Figure 2:**
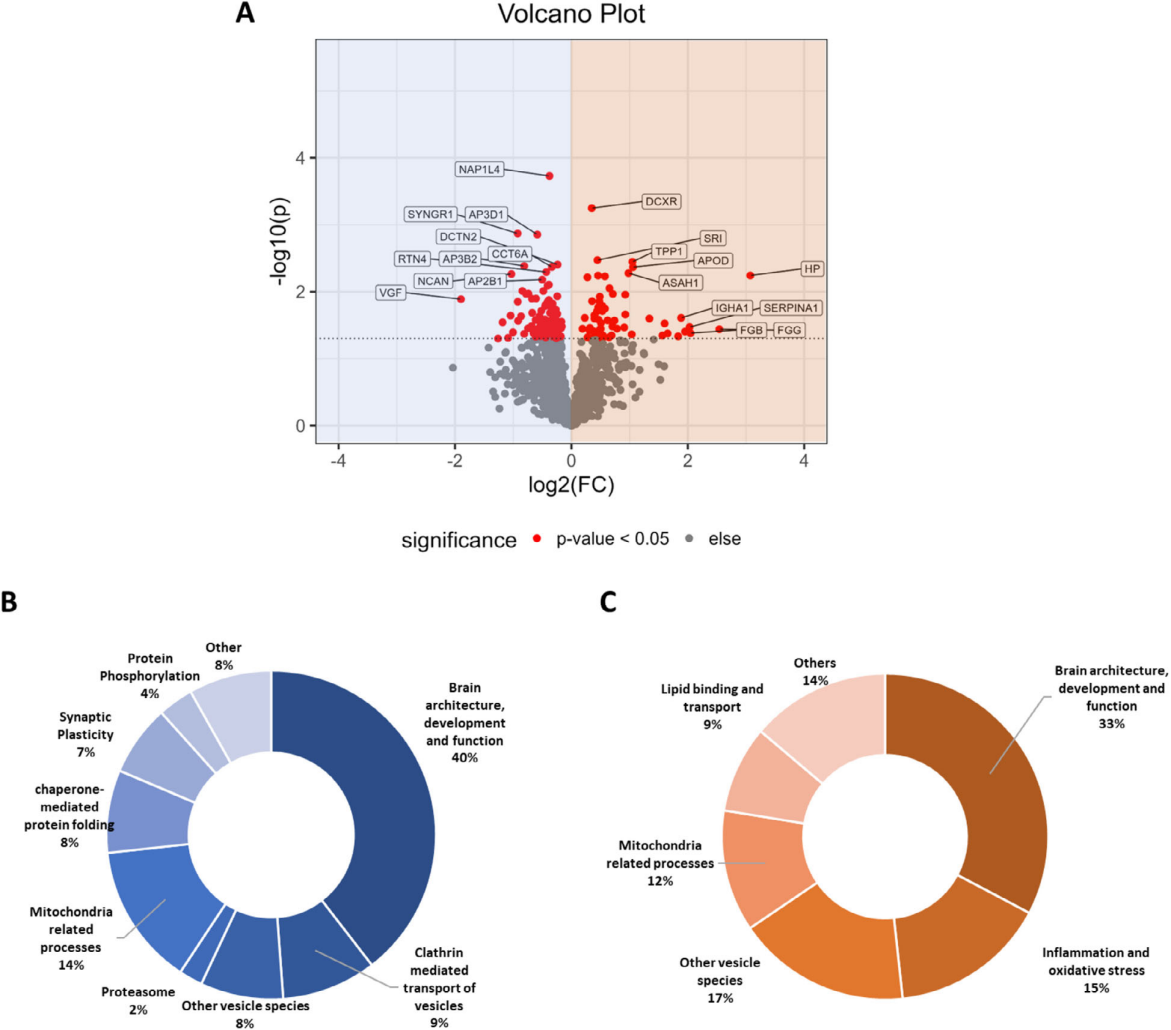
Neuroinflammation‐ and Clathrin‐mediated vesicle transport‐associated proteins are significantly changed between both age groups in the substantia nigra tissue. A) Volcano Plot displaying the differentially abundant proteins (red) between young (highlighted in blue) and old substantia nigra samples (highlighted in orange) deprived of neuromelanin granules (SN_Surr._). B) Manual curation based on Gene Ontology (GO) annotation (based on UniProt entries) of all proteins being of higher abundance in young SN_Surr._ tissue. C) Manual curation based on GO annotation (based on UniProt entries) of all proteins being of higher abundance in old SN_Surr._ tissue.

Since the abovementioned results already indicated the cellular composition to be influenced by age, we hypothesized that aging will also shape the global protein pattern of SN_Surr._ samples. Confirmatively, we identified 144 proteins as changed in abundance in response to aging, whereby 86 proteins were found to be of higher abundance in the young cohort, and 58 proteins were found to be of higher abundance in the old cohort (see **Figure** **2**A; Supplementary Material S2 (Supporting Information); unpaired Student's t‐test *p*‐value < 0.05). To further explore this finding, we conducted an in‐depth analysis of the top 10 proteins based on their Euclidian distance (see **Table** **1**). In the young cohort, we observed that proteins associated with neurogenesis, neurite growth, and vesicle transport were among the most significantly upregulated. Notably, in the old cohort, four proteins included in the top 10 were classified as acute‐phase proteins, which are stimulated during immune reactions and inflammation. These findings were further validated by manual gene ontology (GO) curation (based on UniProt entries) of all differential proteins (see Figure 2B,C; Supplementary Material S3, Supporting Information)

**Table 1:** Top 10 differentially expressed proteins (indicated by protein name and gene name) displaying the highest Euclidian distance in young SN_Surr._ tissue (left, *n* = 4, mean age: 27.8 years) and old SN_Surr._ tissue (right, *n* = 4, mean age: 76 years). Enrichment is indicated by fold change (FC) and significance via *p*‐value (unpaired Student's t‐test *p*‐value < 0.05).

Top 10 proteins being of higher abundance in young SN_Surr._ tissue			Top 10 proteins being of higher abundance in old SN_Surr._ tissue		
Protein name (*Gene name*)	FC young/old	*p*‐value	Protein name (*Gene name*)	FC old/young	*p*‐value
Neurosecretory protein VGF (*VGF*)	3.7	0.0129	Haptoglobin (*HP*)	8.4	0.0057
Neurocan core protein (*NCAN*)	2.0	0.0054	Fibrinogen gamma chain (*FGG*)	5.8	0.0362
Synaptogyrin‐1 (*SYNGR1*)	1.9	0.0013	Fibrinogen beta chain (*FGB*)	4.1	0.0415
AP‐3 complex subunit beta‐2 (*AP3B2*)	1.8	0.0041	Alpha‐1‐antitrypsin (*SERPINA1*)	4.1	0.0337
AP‐3 complex subunit delta‐1 (*AP3D1*)	1.5	0.0013	Ig alpha‐1 chain C region (*IGHA1*)	3.7	0.0246
AP‐2 complex subunit beta (*AP2B1*)	1.4	0.0066	Apolipoprotein‐D (*APOD*)	2.1	0.0043
Reticulon‐4 (*RTN4*)	1.3	0.0051	Tripeptidyl‐peptidase 1 (*TPP1*)	2.1	0.0036
Nucleosome assembly protein 1‐like 4 (*NAP1L4*)	1.3	0.0002	Acid ceramidase (*ASAH1*)	2.0	0.0053
T‐complex protein 1 subunit zeta (*CCT6A*)	1.3	0.0043	Sorcin (*SRI*)	1.4	0.0034
Dynactin subunit 2 (*DCTN2*)	1.2	0.0039	L‐xylulose reductase (*DCXR*)	1.3	0.0005

According to our data, young SN_Surr._ tissue is mainly characterized by proteins essential for brain architecture, development, and function (40%), synaptic plasticity (7%), clathrin‐mediated transport of vesicles (9%), as well as various processes based inside the mitochondrion (14%). Additionally, vesicular proteins as well as proteins of vesicle‐associated organelle species, such as endosomes, exosomes, and lysosomes, were found to be of higher abundance in the young SN_Surr._ (8%). Interestingly, proteins necessary for protein folding and phosphorylation accounted for a high percentage of differentially regulated proteins (12%) within our young cohort (see Figure 2B). These findings were further corroborated by GO‐term enrichment analysis on the basis of cellular compartments (CC), in which, amongst others, terms such as perineuronal net, presynaptic endocytic zone membrane, clathrin coat, and respiratory chain were found to be enriched (see Supplementary Material S3, Supporting Information).

In contrast, only 33% of proteins displaying an increased abundance in the SN_Surr._ of the old cohort could be annotated to essential brain functions; similarly, mitochondria‐related processes only accounted for 12% of all differential proteins. However, the aged SN_Surr._ displayed an enhanced abundance of other vesicle species and related organelles (17%) and was mainly characterized by its high abundance of inflammatory proteins (15%) and an increase in lipid binding and lipid transport (9%) (see Figure 2C). Again, GO‐term enrichment analysis further supported these results, since terms, such as fibrinogen complex, blood microparticle, melanosome, and chromaffin granule membrane were found to be enriched (see Supplementary Material S3, Supporting Information).

Consequently, we were encouraged to undertake a comprehensive examination of protein dynamics associated with neuroinflammation and clathrin‐mediated vesicle transport, as these two processes were found to be particularly altered due to aging. The objective was to ascertain additional information on aging‐related alterations of the human SNpc.

### 2.3 Neuroinflammatory Processes are Apparent in the Aging SNpc

Inflammation is tightly associated with the aging process of the brain and a pathological hallmark of a variety of neurodegenerative diseases. Since several proteins known to be activated or involved in neuroinflammation were found to be among the Top 10 increased proteins in aged SN_Surr._ tissue (based on Euclidian distance, see Table 1, right), we set out to determine protein expression profiles of 30 well‐known acute phase proteins.^[^ ^25^ ^]^ In total, we identified 10 of those in our dataset, including Haptoglobin (HP), Fibrinogen gamma chain (FGG), Fibrinogen beta chain (FGB), Complement C3 (C3), Serotransferrin (TF), Alpha‐1‐antichymotrypsin (SERPINA3), Alpha‐2‐macroglobulin (A2M), Fibrinogen alpha chain (FGA), Hemopexin (HPX) and Alpha‐1‐antitrypsin (SERPINA1). Allof these showed an enhanced protein abundance in the old cohort (unpaired student's t‐test *p*‐value < 0.05, see supplementary Material S2, Supporting Information).

We additionally confirmed increased neuroinflammation using an immunohistochemical‐based approach by staining HP and SERPINA1 (see Figures S1 and S2, Supporting Information). We further verified co‐localisation of HP with NMGs within our old cohort, using an immunohistochemical‐based approach, supporting the theory of NMGs being a hub for neuroinflammation‐related processes (see Figure S1, Supporting Information, white arrows).

### 2.4 The Clathrin‐Mediated Transport is Altered in Aging SNpc

The clathrin protein interaction network comprises a variety of proteins orchestrating its nucleation and assembly, neck constriction, uncoating, and vesicle transport. A comprehensive review^[^ ^26^ ^]^ was consulted to identify all proteins present in our data set involved in the clathrin‐mediated transport of vesicles. With this approach, we were able to map 14 proteins involved in nucleation and assembly, 4 proteins orchestrating the neck constriction, 2 proteins associated with the uncoating process, and 23 proteins contributing to vesicle scission and movement (see Supplementary Material S4, Supporting Information). Of these 43 proteins, 8 were found to be significantly higher abundant in young SN_Surr._ tissue compared to old SN_Surr._ (unpaired student's t‐test *p*‐value < 0.05, see Supplementary Material S2, Supporting Information). To assess trends in abundances of non‐significant proteins, normalized iBAQ values were taken to receive absolute quantitative values for each protein. These values were subsequently utilized to build ratios of abundances between young and old SN_Surr._ Applying this approach, 10 proteins showed similar absolute abundances (ratio iBAQ values: 0.9–1.1), 23 proteins were of higher absolute abundance in the young‐ and 10 proteins in the old cohort. Although proteins changing in their absolute abundance were distributed uniformly across all processes, differences in the absolute abundances of several protein isoforms were observed. Beyond protein isoforms demonstrating a uniform trend, the family of Phosphatidylinositol‐5‐phosphate 4‐kinase type 2 lipid kinases, WASP family members, and the Coronin family exhibited age‐dependent differences in absolute abundance.

### 2.5 Aged NMGs Accumulate Proteins Essential for Brain Architecture, Dopaminergic Pathways, and Mitochondrial Function

**Figure 3:**
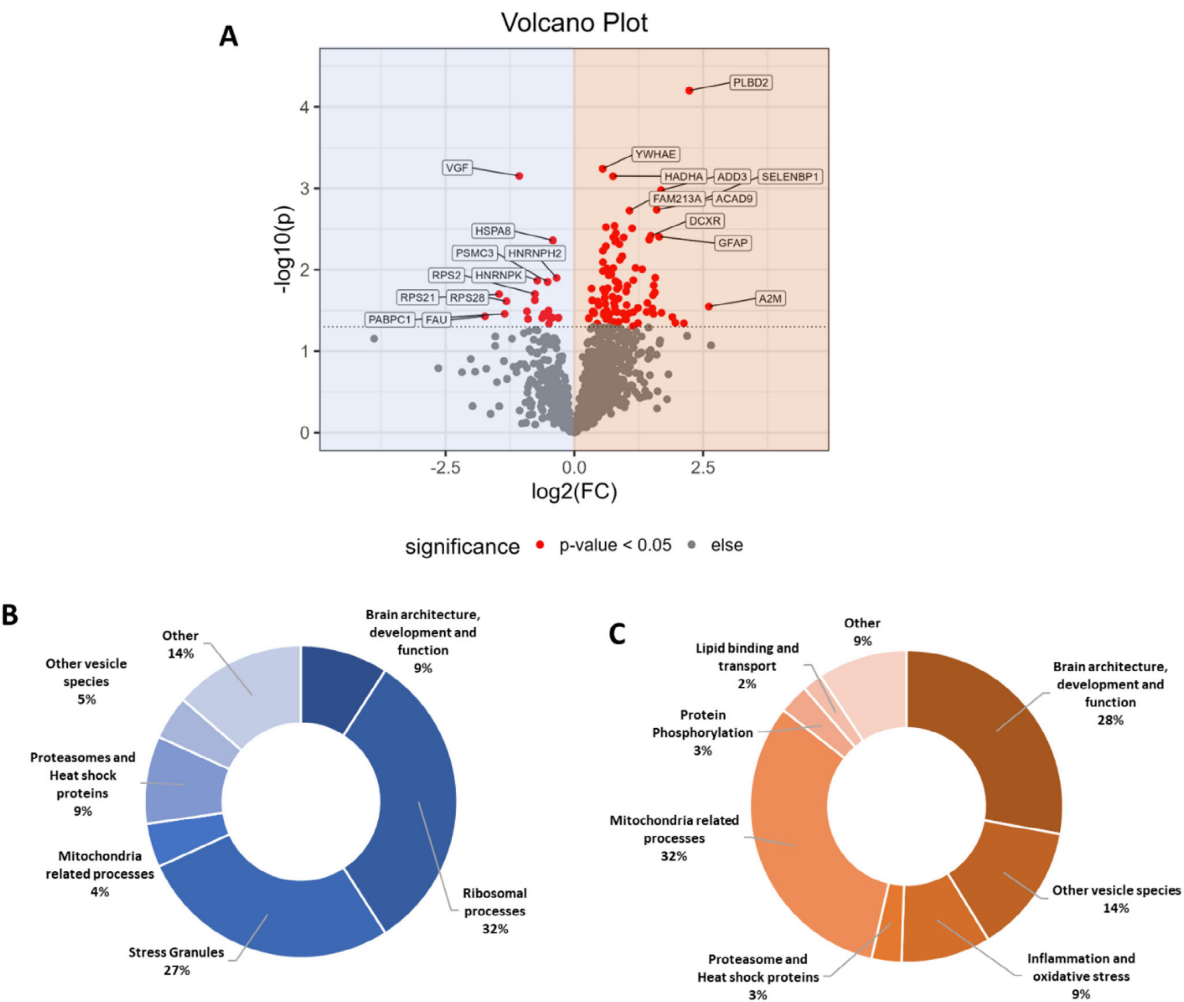
Mitochondrial and ribosomal proteins are altered in abundance between young and old neuromelanin granules (NMGs). A) Volcano Plot displaying the differentially abundant proteins (red) between young (highlighted in blue) and old NMG samples (highlighted in orange). B) Manual curation based on Gene Ontology (GO) annotation (based on UniProt entries) of all proteins being of higher abundance in young NMGs. C) Manual curation based on GO annotation (based on UniProt entries) of all proteins being of higher abundance in old NMGs.

PD is characterized by the depigmentation of the SNpc, as a result of dopaminergic neurons loss containing NMGs. Thus, the spatially resolved analysis of NMGs may facilitate the elucidation of age‐dependent changes tightly related to early PD pathologies. With the aim of identifying proteins being differently regulated in the aging NMG proteome, a relative quantification of all identified proteins was performed (see **Figure** **3**). Out of 1168 proteins, 22 proteins were found to be significantly higher abundant within the young cohort and 97 within the old cohort (unpaired Student's t‐test *p*‐value < 0.05).

In the young cohort, among the Top 10 proteins displaying the highest differences (Euclidian distance), mainly ribosomal proteins, as well as one proteasomal protein, could be identified. To our surprise, again, Neurosecretory protein (VGF) was found to be of higher abundance in the young cohort. In contrast, proteins linked to intra‐Golgi transport and signal transduction, astrocytes, lysosomes, and mitochondria appeared to be elevated in the old cohort (see **Table** **2**). An in‐depth analysis of all differentially expressed proteins was again achieved by manual curation based on GO association (based on Uniprot entries) and GO‐term enrichment analysis (see Figure 3B,C; Supplementary Material S2, Supporting Information). Processes apparent in young NMGs could mainly be annotated to the ribosome (31.8%) and stress granules (27.3%). Confirmatively, GO‐term enrichment analysis resulted in the identification of terms such as small and large ribosomal subunit and cytosolic/polysomal ribosome (see Supplementary Material S3, Supporting Information). The aged NMGs instead were mainly characterized by an increased abundance of proteins associated with mitochondrial (32%) processes or proteins essential for brain architecture, development and function (27.8%), notably Parkinson disease protein 7 (PARK7) was found to be of higher abundance in aged NMGs, a protein genetically linked to PD and involved in the dopaminergic pathway.^[^ ^27^ ^]^ Interestingly, the aged NMG proteome displayed an increase in abundance of four proteins involved in dopaminergic pathways as well: Amine oxidase [flavin‐containing] A (MAOA) and B (MAOB),^[^ ^28^ ^]^ Vacuolar protein sorting‐associated protein 35 (VPS35),^[^ ^29^, ^30^ ^]^ and Cytosolic 10‐formyltetrahydrofolate dehydrogenase (ALDH1A1).^[^ ^31^ ^]^ Additionally, proteins not directly involved in dopaminergic pathways, but known to be associated with Parkinson's disease, Leucine‐rich PPR motif‐containing protein, mitochondrial (LRPPRC),^[^ ^32^ ^]^ Lon protease homolog, mitochondrial (LONP1)^[^ ^33^ ^]^ were found to be significantly upregulated (unpaired student's t‐test *p*‐value < 0.05). In regards to the observed increased neuroinflammation in the aged SN_Surr._, we were not surprised to identify Ferritin, the major iron‐storing protein, to be increased in aged NMGs. Further, different vesicle species, such as exosomes and lysosomes (13.4%) and inflammatory processes, including oxidative stress (9.3%), defined the aged NMG proteome. GO‐term enrichment analysis of differential proteins supported these findings since terms found to be enriched were associated with mitochondria (e.g., mitochondrial pyruvate dehydrogenase complex, mitochondrial matrix and membrane) and lysosomes, as well as different vesicle species (e.g., lysosomal lumen, lysosome, extracellular exosome, extracellular vesicle, synaptic vesicles, see Supplementary Material S3, Supporting Information).

**Table 2:** Top 10 differentially expressed proteins (indicated by protein name and gene name) displaying the highest Euclidian distance in young NMGs (left, *n* = 4, mean age: 27.8 years) and old NMGs (right, *n* = 4, mean age: 76 years). Enrichment is indicated by fold change (FC) and significance via *p*‐value (unpaired Student's t‐test *p*‐value < 0.05).

Top 10 proteins being of higher abundance in young NMGs			Top 10 proteins being of higher abundance in old NMGs		
Protein name (Gene name)	FC young/old	*p*‐value	Protein name (Gene name)	FC old/young	*p*‐value
Ubiquitin‐like FUBI‐ribosomal protein eS30 fusion protein (*FAU*)	3.3	0.0372	Alpha‐2‐macroglobulin (*A2M*)	6.1	0.0282
Small ribosomal subunit protein eS21 (*RPS21*)	2.8	0.0199	Selenium‐binding protein 1 (*SELENBP1*)	5.4	0.0014
Polyadenylate‐binding protein 1 (*PABPC1*)	2.6	0.0348	Putative phospholipase B‐like 2 (*PLBD2*)	4.7	0.0000
Small ribosomal subunit protein eS28 (R*PS28*)	2.5	0.0244	Gamma‐adducin (*ADD3*)	3.2	0.0011
Neurosecretory protein VGF (*VGF*)	2.1	0.0007	Glial fibrillary acidic protein (*GFAP*)	3.1	0.0039
Small ribosomal subunit protein uS5 (*RPS2*)	1.7	0.0199	Acyl‐CoA dehydrogenase family member 9 (*ACAD9*)	3.0	0.0018
Heterogeneous nuclear ribonucleoprotein K (*HNRNPK*)	1.6	0.0136	L‐xylulose reductase (*DCXR*)	2.8	0.0038
26S proteasome regulatory subunit 6A (*PSMC3*)	1.4	0.0141	Redox‐regulatory protein FAM213A (*FAM213A*)	2.1	0.0019
Heat shock cognate 71 kDa protein (*HSPA8*)	1.3	0.0044	Trifunctional enzyme subunit alpha (*HADHA*)	1.7	0.0007
Heterogeneous nuclear ribonucleoprotein (*H2HNRNPH2*)	1.3	0.0126	14‐3‐3 protein epsilon (*YWHAE*)	1.5	0.0006

Summarising, the spatially resolved analysis resulted in the identification of several characteristics of aging NMGs, including an altered abundance of mitochondrial, stress granule, and ribosomal proteins. In order to provide further information on potential alterations caused by associated proteins, the focus was directed toward all quantified proteins associated with said proteins.

### 2.6 Unique Alterations in Mitochondrial Proteins can be Observed in the Aging SNpc and aged NMGs

In general, mitochondrial alterations and pathologies are common hallmarks of both aging and neurodegenerative diseases. Our analysis provided us with a spatially resolved view describing an increase in abundance of mitochondria‐associated proteins in young SN_Surr._ tissue compared to aged SN_Surr._ (see Supplementary Material S2, Supporting Information), 12 proteins, unpaired student's t‐test *p*‐value < 0.05), but a reversed regulation in NMG, whereby a higher abundance of mitochondrial proteins was observed in aged NMG compared to young NMG (see Supplementary Material S2, Supporting Information, 31 proteins, unpaired student's t‐test *p*‐value < 0.05). Interestingly, mitochondrial proteins that are of higher abundance in young SN_Surr._ tissue could be mainly annotated to the mitochondrial respiratory chain, whereby proteins displaying the highest fold changes in old NMGs were found to be associated with the fatty acid beta oxidation.

To complete the picture, we aimed to assess the abundance of all quantified mitochondrial proteins on the basis of absolute protein values (normalized iBAQ values). To elucidate this, we utilized the MitoCarta 3.0 database^[^ ^34^ ^]^ and extracted the Top 100 mitochondria‐associated proteins, of whose 62 were present in SN_Surr._ tissue and 65 in NMGs (see Supplementary Material S5, Supporting Information).

**Figure 4:**
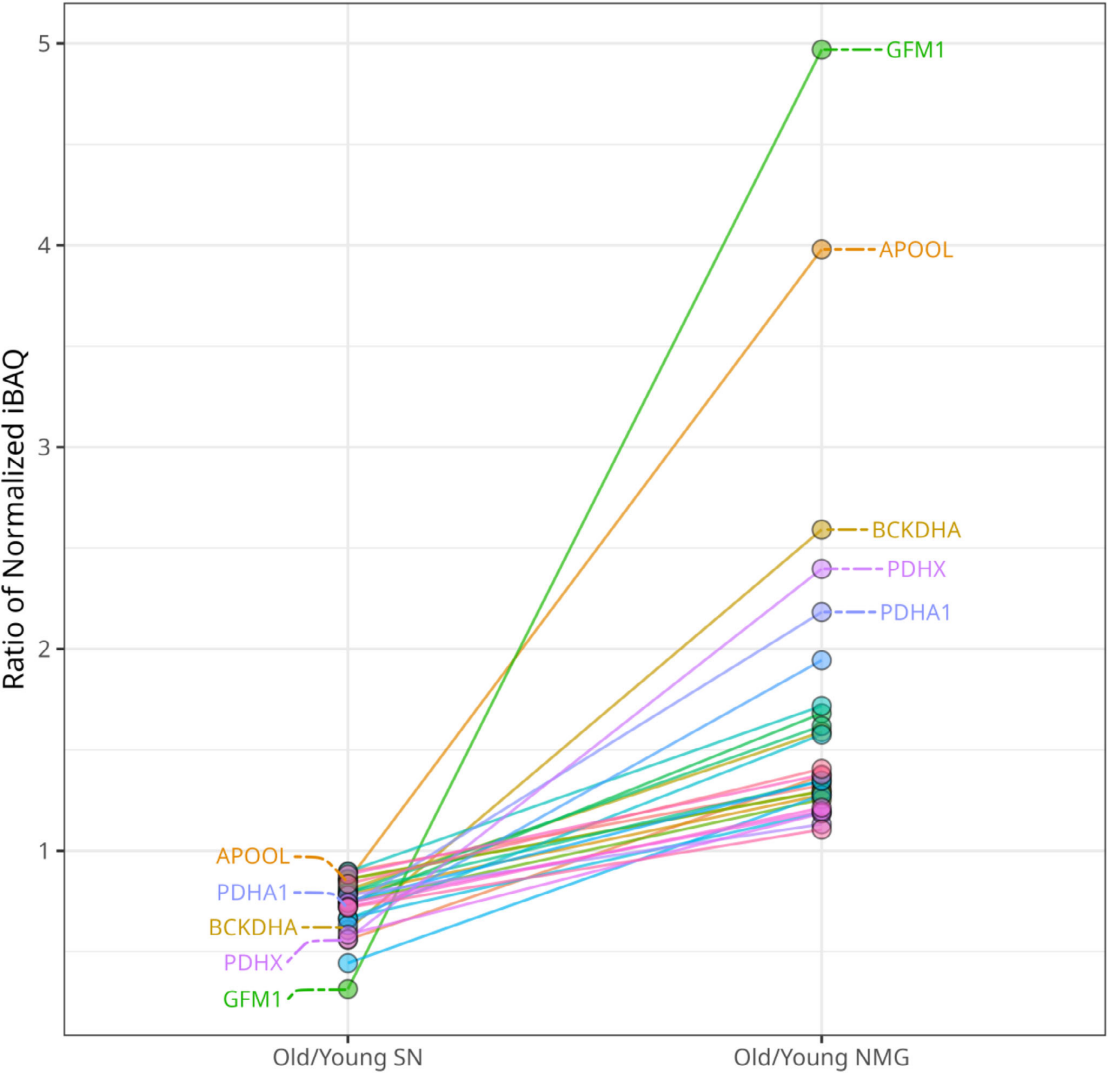
Selected mitochondrial proteins display an increased abundance in aged NMGs. Mitochondrial proteins displaying a discrepancy in their absolute abundance ratio between old (*n* = 4, mean age: 76 years) and young SN_Surr._ tissue (*n* = 4, mean age: 27.8 years) and old (*n* = 4, mean age: 76 years), and young NMGs (*n* = 4, mean age: 27.8 years) were assessed on the basis of normalized iBAQ values. The five proteins with the highest changes in ratios between the comparisons of old/young SN_Surr._ and Old/Young NMG are marked with gene names. Abbreviations: Elongation factor G, mitochondrial (GFM1), MICOS complex subunit MIC27 (APOOL), 2‐oxoisovalerate dehydrogenase subunit alpha, mitochondrial (BCKDHA), Pyruvate dehydrogenase protein X component, mitochondrial (PDHX), and Pyruvate dehydrogenase E1 component subunit alpha, somatic form, mitochondrial (PDHA1).

Of 62 mitochondria‐associated proteins present in SN_Surr._ tissue, 46 showed a higher absolute abundance in young SN_Surr._ tissue, 7 remained unchanged (ratio normalized iBAQ values 0.9‐1.1), and 9 displayed higher absolute abundances in the aged SN_Surr._ In NMGs, instead, out of 65 mitochondria‐related proteins, 44 proteins appeared to be of higher absolute abundance in the old cohort, 13 remained unchanged, and 8 proteins exhibited higher iBAQ ratios in the young cohort. To this end, a cross‐comparison of the relevant proteins was conducted. Out of the 46 proteins of higher abundance in young SN_Surr._ tissue and 44 proteins being of higher abundance in old NMGs, 29 proteins were present in both data sets. Of those, 9 proteins displayed a high discrepancy in ratio, whereby an increased abundance in young SN_Surr._ tissue compared to aged SN_Surr._ tissue, but a decreased abundance in young NMG versus aged NMGs was observed (see **Figure** **4**).

### 2.7 Ribosomal‐ and Stress Granule‐Associated Proteins show a Spatial and Age‐Dependent Enrichment in Young NMG

The relative comparison between young and old NMGs revealed 12 stress granule‐ and 7 ribosome‐associated proteins to be of higher abundance in the young cohort (unpaired student's t‐test *p*‐value < 0.05). Thus, again, we investigated the abundance of all identified ribosomal and stress granule proteins in our dataset on the basis of normalized iBAQ values.

**Figure 5:**
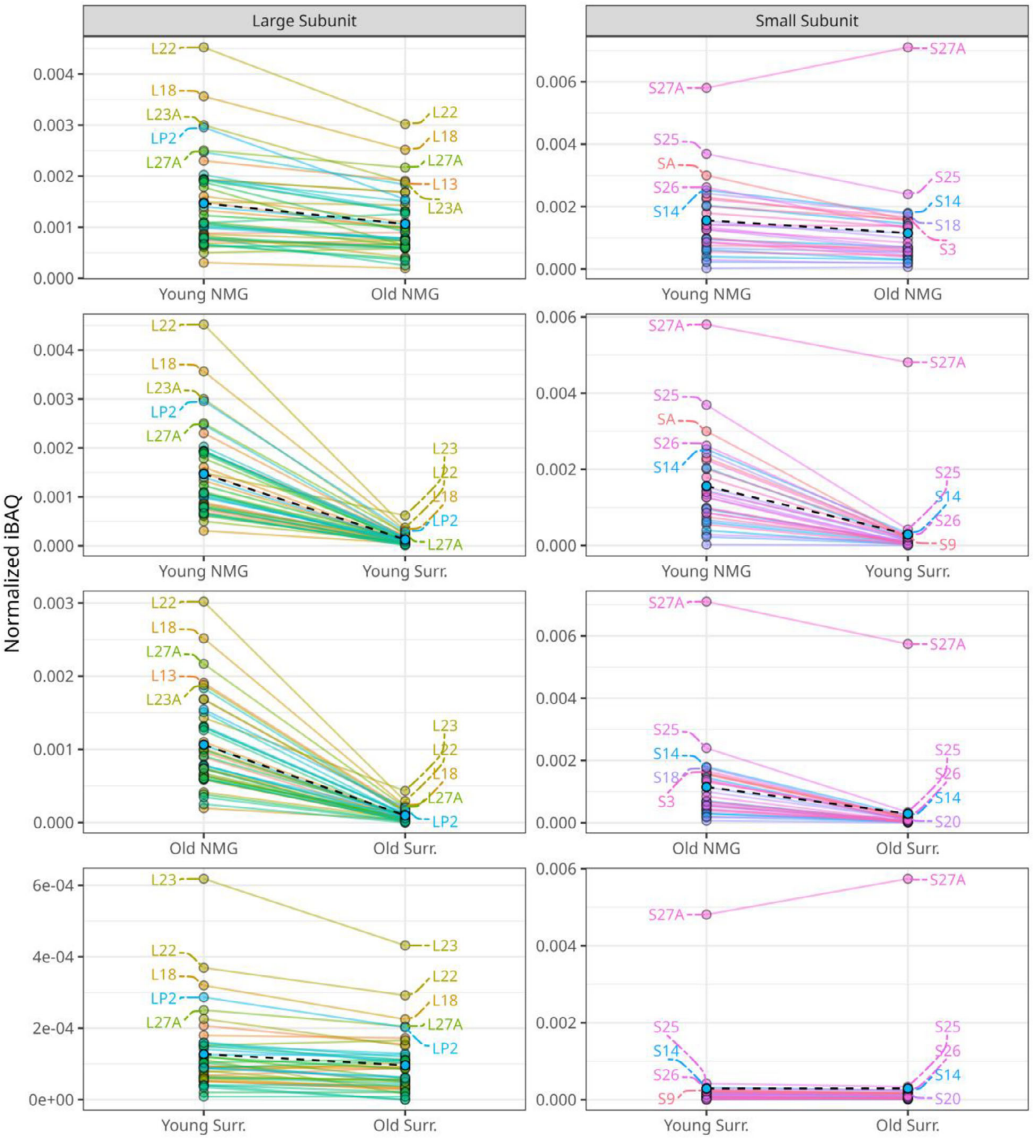
Abundances of ribosomal proteins are mildly affected by aging, but their localization to NMGs is highly specific. The absolute abundance of all proteins of the small and large ribosomal subunits was calculated by normalized iBAQ values. Between young and old NMG or SN_Surr._ tissue a slight decrease in abundance of several large ribosomal subunits can be observed. Further, it is evident that a higher absolute abundance of both subunits is present in all NMG samples compared to SN_Surr._ tissue regardless of age. The mean absolute abundance of all selected proteins is visualized as a dotted line.

Based on 722 proteins identified as being associated with stress granules,^[^ ^35^, ^36^ ^]^ 262 proteins were present in our NMG dataset, 256 in young NMGs, and 252 in old NMGs. Of those proteins, 9 were solely quantified in young NMGs, 162 were found to have a higher absolute abundance in young NMGs (ratio iBAQ values > 1.1), 36 remained unchanged between young and old NMGs (ratio iBAQ protein values 0.9–1.1), and 59 displayed higher values in old NMGs (see Supplementary Material S6, Supporting Information), supporting the hypothesis of an increased abundance of SG proteins in young NMGs. Similarly, all proteins associated with the large ribosomal subunit (43 proteins in total) and 31 out of 32 proteins of the small ribosomal subunit showed a higher absolute abundance in young NMGs (see Supplementary Material S7, Supporting Information). Solely, the small ribosomal subunit S27A displayed a different direction of abundance in both age comparisons. In order to support our previous investigations, we additionally plotted calculated normalized iBAQ values of all ribosomal subunits for both sample types, NMG and SN_Surr._ (see **Figure** **5**). This enabled us to confirm a general higher absolute abundance of ribosomal subunits in NMGs when compared to SN_Surr._, leading to the assumption of a spatial‐specific enrichment (see Figure 5). An age‐dependent effect could be observed for a few proteins of the large subunits, in particular large ribosomal subunit proteins L22, L18, L23A, and LP2, which all showed a lower absolute abundance from young to old NMG and SN_Surr._ tissue. Finally, we sought to exclude the possibility of cross‐contamination of our NMG samples by nuclei or endoplasmic reticulum (ER), based on their close spatial proximity to NMGs. To do so, we elucidated the abundance of nuclei‐ and ER‐marker proteins, confirming a comparable absolute abundance of nuclei‐associated proteins in NMG and SN_Surr._ (see Figure S3 and Supplementary Material S8, Supporting Information). Consequently, the elevated presence of ribosomal proteins in the NMGs can be regarded as a reliable finding.

## 3 Discussion

With our exploratory study investigating region‐specific age‐related changes within the human SNpc in a spatially resolved manner, we are one of the first to provide evidence on age‐dependent molecular alterations in said brain region, which are also known to be key pathologies in neurodegeneration. By investigating the human SNpc, we laid an emphasis on PD, since this brain region is known to be particularly vulnerable due to its high content of dopaminergic neurons. In future studies, the investigation of additional brain regions, such as locus coeruleus, as it is thought to be affected first in PD, or the cortex and hippocampus, which both are primarily affected in Alzheimer's disease, would be of highest interest to study region‐specific alterations as well as communalities and singularities during the aging process. Additionally, orthogonal techniques such as Nanostring or Xenium may in the future allow for verifying and enlarging LMD‐based results. These novel techniques are gaining popularity to study spatially resolved changes in the human brain.^[^ ^37^, ^38^ ^]^ Since both techniques are primarily designed to investigate the transcriptome, single‐cell approaches will become feasible, which so far is not possible on the level of spatial tissue proteomics. However, to our knowledge, only a few studies utilizing said techniques to study the aging brain have been published so far.^[^ ^39^, ^40^, ^41^ ^]^ For the SNpc in particular, no publication was found employing said techniques.

Utilizing our spatial LMD and mass spectrometry‐based workflow, we were able to confirm:

- The specific decrease of marker proteins for dopaminergic neurons and proteins associated with the dopamine metabolism in the SN_Surr._ of elderly subjects.
- An increase in astrocytic proteins in the aged SN_Surr._
- An increase in inflammatory processes in aging SN_Surr._ in proximity to NMGs, as well as an altered vesicle trafficking machinery in NMGs.
- Unique age‐dependent alterations of mitochondrial proteins in aged SN_Surr._ tissue and NMGs
- The overall high abundance of ribosomal and stress granule‐associated proteins in NMGs compared to SN_Surr._ tissue, with higher abundances in young NMGs compared to those of older tissue donors.

These major findings will be discussed in detail below.

### 3.1 Proteomic Profiling of Aging SNpc Tissue Points Toward an Age‐Dependent Loss of Dopaminergic Neurons

SNpc tissue has been previously described as being mainly composed of neurons, astrocytes, and oligodendrocytes.^[^ ^23^ ^]^ When comparing the protein abundances of marker proteins for these cell types, we found general neuronal markers (NEFL, NEFM, NEFH, APP) to remain stable in their abundance, with the exception of MAPT and SNCA, which showed a slight decrease, respectively 47% and 45% in the aged cohort. Furthermore, marker proteins for dopaminergic neurons (TH, DDC, SLC6A3) demonstrated a more pronounced decrease, suggesting an age‐dependent loss of dopaminergic neurons in the SNpc or reduced protein levels in surviving dopaminergic neurons. This decline has been corroborated by several other studies, as reviewed by Eriksen et al.^[^ ^42^ ^]^ In addition, mild to moderate reductions in the number of dopaminergic neurons of the SNpc as well as dopamine receptor levels with physiological aging were reported in a review by Trist et al.,^[^ ^43^ ^]^ in which the researchers pointed out that oxidative stress in the aging SNpc may be a key factor underlying its “selective vulnerability”. Another potential explanation is the hypothesized loss of the dopaminergic phenotype of respective neurons in the SNpc, once a certain intracellular threshold of neuromelanin concentration is reached.^[^ ^19^, ^44^, ^45^ ^]^ In contrast to that, the aged NMG proteome displayed an enrichment of proteins known to be associated with the dopaminergic pathway and PD: MAOA, MAOB, VPS35, and ALDH1A1, LRPPRC, and LONP1. Two of those proteins were found to be differential in our previous study as well, whereby MAOB displayed a slight increase in elderly subjects, and VPS35 a slight decrease in elderly subjects. Since VPS35 is known for transporting cargo between different vesicle species, one might assume that the accumulation of VPS35 in aged NMG may be a result of NMGs proposed role as a lysosomal organelle.

Astrocytic proteins (GFAP, S100B) were found to show an increase in abundance with age, which may either indicate an increase in astrocytic activity or an increase in the number of astrocytes in the SNpc, the latter of which was already verified via immunohistochemistry by directly connecting neuronal loss with an increase in astrocyte numbers.^[^ ^16^ ^]^

Oligodendrocytes appeared to be relatively unresponsive to the effects of aging, as evidenced by the stability in the levels of MBP and PLP1 between the young and old cohorts. This finding is consistent with a study that utilised post‐mortem brain tissue and immunohistochemical staining of CNP, which revealed only subtle demyelination of the SNpc. This was interpreted as indicative of oligodendrocytes maintaining their functionality during physiological aging.^[^ ^16^ ^]^

### 3.2 Neuroinflammatory Responses are a Key Hallmark of the Aging SNpc and are Detectable in Aged NMGs as well

Neuroinflammation is thought to be a major contributing factor to various abnormalities associated with physiological brain aging, as well as in cases of injury, infection, stress, and diseases.^[^ ^46^, ^47^ ^]^ For PD in particular, neuroinflammation is thought to play a pivotal role,^[^ ^48^, ^49^ ^]^ whereby elevated levels of proinflammatory cytokines,^[^ ^50^ ^]^ as well as changes in the adaptive immune response^[^ ^51^ ^]^ were frequently observed in blood samples of patients. In confirmation with our previous study,^[^ ^21^ ^]^ we identified several proteins associated with inflammatory processes to be of higher abundance in SN_Surr._ tissue of aged individuals (unpaired student's t‐test *p*‐value < 0.05), among them HP (FC 8.4), FGG (FC 5.8), FGB (FC 4.1), SERPINA1 (FC 4.1), and C3 (FC 3.9). By immunohistochemistry, we and others were able to show that NMGs are a major hub for neuroinflammation in the aged SNpc (see Figure S1, Supporting Information),^[^ ^15^, ^16^ ^]^ potentially explaining the postulated bivalent neuroprotective and neurotoxic function of NMGs, by storing molecules causing oxidative stress, a hypothesis that is further substantiated by an increased abundance of PRDX1 and PRDX6, both proteins essential for redox homeostasis, in old SN_Surr._ tissue.

In conclusion, these findings point towards and confirm neuroinflammation on the basis of proteome analyses as a mechanism associated with aging, potentially based on the oxidative environment present in the human SNpc and a consistently increasing inability to handle the oxidative stress. With our spatially resolved approach, we can substantiate that during this process, NMGs seem to act as hubs of neuroinflammation, since inflammation‐associated proteins are highly abundant in the direct surrounding.

### 3.3 The Clathrin Interaction Network Shows Age‐ and Region‐Specific Alterations

In the context of the central nervous system, clathrin‐mediated endocytosis plays a pivotal role in the process of recycling synaptic vesicles in presynaptic terminals and in the recycling of transmitter receptors in neuronal somata/dendrites. This significant endocytic system has been observed to be compromised in the context of neurodegenerative diseases, including Alzheimer`s disease^[^ ^52^, ^53^, ^54^ ^]^ and PD.^[^ ^55^ ^]^ In the latter, genetic forms of the disease have been linked to mutations in vesicular trafficking. Still, studies on the abundance of clathrin‐associated proteins in the context of aging are rare. In this study, we present the first comprehensive analysis of protein expression levels of clathrin‐associated proteins in the SNpc, thereby providing a spatially resolved perspective on clathrin dynamics. Utilising a mass spectrometric approach, we have identified 43 proteins within our dataset that are associated with clathrin‐mediated vesicle transport, of which 23 proteins exhibited higher abundance levels in the younger cohort and 10 proteins in the older cohort (based on normalized iBAQ values).

In contrast to a former study by Alsquati and colleagues^[^ ^56^ ^]^ which showed a trend towards a higher abundance of clathrin‐related proteins in the aged human cortex, clathrin showed a significant enrichment in our young subjects, likely to Dynamin‐1 and the Dynamin‐1‐like protein, potentially indicating a spatial difference in the level of clathrin‐associated proteins in the aging SNpc. Interestingly, as already supported in the abovementioned study, differences in the expression profiles of several protein isoforms were observed in our cohort. Besides protein isoforms which showed a uniform trend, such as Profilin1&2 (higher in old SN_Surr._), the Coronin (CORO) family among others showed an age‐dependent expression profile, with enhanced expression of CORO1A in the young cohort, an equal expression of CORO1B and CORO1C and higher expression profiles of CORO2A as well as CORO2B in the old cohort. Encouraged by these results, we investigated the role of these isoforms, potentially explaining their adverse behaviour. Indeed, for several isoforms (in)direct connections to age‐related alterations could be formulated. CORO2A, e.g., is known to mediate the actin‐dependent de‐repression of inflammatory response genes^[^ ^57^ ^]^ and may therefore promote inflammatory processes in the aging SNpc. In addition, profilins have been demonstrated to be overexpressed in response to processes that promote the production of reactive oxygen species, such as the AGE receptor (RAGE), activating Protein kinase C and NF‐κB pathways, in Alzheimer's disease^[^ ^58^ ^]^ and patients experiencing hyperglycaemia,^[^ ^59^, ^60^ ^]^ thereby inducing an inflammatory response,

To summarise the findings on the abundance levels of proteins associated with clathrin‐mediated transport in response to aging, it is evident that a definitive conclusion cannot be drawn at this time. The seemingly contradictory findings of the aforementioned studies and our own data initiate a discussion on the subject of spatially resolved differences in protein expression levels in the brain. Indeed, recent studies involving the analysis of transcripts in specific regions of the mouse brain lend support to the aforementioned hypothesis of spatial differences in protein abundances. Here authors pointed out that at least 82 genes are differentially regulated in 10 or more brain regions. All 82 genes compromised immune‐modulatory functions, including MHC I‐mediated antigen presentation, interferon‐response, cell adhesion, and activation of the complement cascade, as well as regulators of mouse microglia activity.^[^ ^61^ ^]^ Authors concluded that the impact of aging on brain function is specific, potentially even related to the regional vulnerability across different diseases, as well as the varied manifestations in individual patients. Therefore, it will be important to consider the spatial component in future studies focusing on the proteome of the aging brain.

### 3.4 Changes in Expression in Mitochondrial Proteins Potentially Indicate an Age‐Dependent Shift in Localisation and Support the Theory of NMGs Being of Lysosomal Offspring

Mitochondrial alterations are a well‐known hallmark of aging and a variety of neurodegenerative diseases. Mutations and deletions in mtDNA, morphological alterations in shape and size, as well as an altered expression of mitochondrial proteins have been observed. A recent study further verified an overall down‐regulation of mitochondrial processes by analysing different transcriptome signatures in the mouse brain, whereby especially the aged striatum showed a decreased expression of mitochondrial‐associated proteins,^[^ ^61^ ^]^ indicating spatially resolved differences within brain regions as reported for the clathrin‐related proteins. Consequently, the detection of a decreased abundance of mitochondria‐associated proteins in aged SN_Surr._ tissue was not unexpected, thus supporting the hypothesis of a mitochondrial loss during the process of aging. However, when analysing aged NMGs, a distinct accumulation of these proteins could be identified, which could cautiously be interpreted as an age‐driven location shift in mitochondrial proteins from SNpc tissue into NMG structures. Out of 45 proteins being of higher abundance in young SN_Surr._ tissue and 44 proteins being of higher abundance in old NMGs, 28 proteins were present in both sample types, meaning that 62% of proteins were potentially shifted in their location in response to aging.

This phenomenon may be explained by the nature of NMGs themselves. Several studies analysing their morphological structure and protein content concluded that they may act as a specific form of lysosomes in dopaminergic neurons, as they are surrounded by a double membrane, a classic organelle feature, and inherit a high abundance of lysosomal proteins.^[^ ^12^, ^23^ ^]^ Consequently, if NMGs are indeed of lysosomal offspring, the increased abundance of mitochondria in aged NMGs, but not in aged SN_Surr._ tissue may be a result of macroautophagic processes trying to remove defective mitochondria in the aging SNpc. Supporting this hypothesis, we were able to identify PARK7, Non‐selective voltage‐gated ion channel VDAC1 and 2 (VDAC1, VDAC2), and LONP1 being of higher abundance in aged NMGs (unpaired student's t‐test *p*‐value < 0.05). All are involved in protecting mitochondrial function, e.g., dysfunctional PARK7 leads to mitochondrial defects^[^ ^62^ ^]^ whereby VDAC1 is known to promote mitophagy in depolarized mitochondria^[^ ^63^ ^]^ and VDAC2 is thought to have a protective effect in cells undergoing mitochondrial apoptosis during aging.^[^ ^64^, ^65^ ^]^ Further, we identified LONP1 as being of higher abundance in aged NMGs, an ATP‐dependent serine protease, mediating the selective degradation of misfolded, unassembled, or oxidatively damaged proteins in the mitochondrial matrix and participating in the regulation of mitochondrial gene expression. Indeed, LONP1 was additionally suspected to play a direct role in the pathogenesis of PD by participating in the degradation of unstable PD‐associated DJ‐1/PARK 7 missense mutants.^[^ ^33^ ^]^ Notwithstanding, given the present methods and samples, it remains unconfirmed whether these proteins undergo active translocation into NMGs. Further, it cannot be concluded whether either single mitochondrial proteins originating from defective mitochondria are transported into NMGs, or whether defective mitochondria are imported into NMGs for degradation directly. In theory, age‐associated changes in energy demand may also result in a decreased abundance of mitochondrial proteins in aging SN_Surr._ tissue, which may further explain an increased degradation of mitochondria. Nevertheless, this cautious hypothesis would substantiate the proposed relation between lysosomes and NMGs.

### 3.5 Stress Granule Abundance is Tightly Connected to Aging and Plays a Role in Age‐Dependent Alterations in the Human Brain

Stress granules (SGs) are transient membrane‐less organelles that are formed by RNA‐binding proteins and RNA during acute cell stress. The formation of SGs is thought to serve to protect unstable RNA species under cell stress, contributing to rapid cell recovery. However, in recent years, the process of SG formation was found to be altered in diseases associated with stress and inflammation, such as cancer,^[^ ^66^ ^]^ viral infection,^[^ ^67^, ^68^ ^]^ and neurodegenerative diseases.^[^ ^69^, ^70^, ^71^ ^]^ Also in the context of healthy aging, SGs have been demonstrated to play a significant role, as cellular senescence has been found to impair the correct formation of SGs in kidney cells.^[^ ^72^ ^]^ A secondary study has corroborated the finding that cellular senescence impedes the proper formation of SGs, whereby exposure to stress could induce the formation of SGs in proliferating cells, but not in fully senescent human fibroblasts.^[^ ^73^ ^]^ Confirmatively with our data, loss of G3BP1 in a KO mouse model leads to alterations in the control of neuronal plasticity and calcium homeostasis, stressing its immense importance in neural cells.^[^ ^74^ ^]^ In our last study, we found evidence for a general enrichment of SG proteins within NMGs, when compared to SN_Surr._ tissue, leading us to the cautious hypothesis that SGs may either be involved in NMG formation, form in close proximity to NMGs as a result of enhanced oxidative stress caused by neuromelanin‐bound metals, or to stabilize the NMG membrane, since SGs were observed to rapidly form at damaged endomembrane sites.^[^ ^75^ ^]^

Confirming prior results in aging SNpc tissue,^[^ ^21^ ^]^ in which SG‐related proteins were found to be increased in young individuals, with our current study, we are now able to verify an increased abundance of SG‐related proteins in NMGs of our young cohort, supporting the recent findings on SG alterations in aging. Of those six proteins found to be of higher abundance in young NMGs (student's t‐test *p*‐value < 0.05), three were Heterogeneous nuclear ribonucleoproteins, which are known to bind pre‐mRNA and to regulate neuronal differentiation. Further, Polyadenylate‐binding protein 1 (PABPC1), a stress granule assembly protein, was found to be significantly enriched.

We hypothesize that the assembly and disassembly of SG structures may be hampered during the aging processes, as the majority of SG proteins involved in assembly,^[^ ^76^, ^77^ ^]^ among them Caprin‐1 (CAPRIN1), Ras GTPase‐activating protein‐binding protein 1 and 2 (G3BP1/G3BP2), and Polyadenylate‐binding protein 1 (PABPC1) and in disassembly,^[^ ^76^, ^77^ ^]^ Ubiquitin carboxyl‐terminal hydrolase 10 (USP10) and Ubiquitin‐associated protein 2‐like (UBAP2L) were found to be decreased in abundance in NMGs of old tissue donors (normalized iBAQ ratios < 0.9). We further identified the complete absence of DHX36^[^ ^78^ ^]^ in old NMGs, a helicase unwinding G4 RNAs, which is thought to act as an essential factor in SG assembly. Confirmatively, we identified DHX30 as being increased in young NMG, which is thought to have a similar function.^[^ ^79^ ^]^ Thus, we conclude that the reduced abundance or absence of SG components in aged subjects may hint at a defective stress response and thus the accumulation of oxidative stress and enhanced inflammation within brain tissue, as supported by an increase in acute phase proteins. This hypothesis is in line with the observation that persistent SGs are thought to be the origin of abnormal protein aggregates in neurodegenerative diseases, as has been proposed before. ^[^ ^70^, ^80^, ^81^ ^]^

### 3.6 The High Abundance of Ribosomal Proteins within NMGs Opens a New Discussion about NMG Biology

The present study builds on the findings of a previous study,^[^ ^23^ ^]^ which demonstrated that NMGs exhibit an enhanced abundance of the large and small ribosomal subunits compared to SN_Surr._ tissue in healthy aged individuals. This observation has the potential to contribute to our understanding of NMG biology by highlighting its role in protein translation. However, the hypothesized connection of lysosomes and NMGs may be the major cause of ribosomal accumulation, as ribosome biogenesis, as well as their stoichiometry and their translation efficiency, were found to be altered as a response to aging.^[^ ^82^, ^83^, ^84^ ^]^ In our current study, we were able to assess whether aging has an influence on ribosomal location. Indeed, of the 43 quantified proteins associated with the large subunit, all showed enhanced expression profiles in young NMGs compared to their older counterparts, and a similar trend was observed for the small subunit (32 quantified proteins), with one exception: 40S ribosomal protein S27a. This protein contains a covalently attached ubiquitin, potentially functioning in protein degradation via the proteasome. Consequently, the hypothesis that the accumulation of ribosomal proteins in NMGs is attributable to autophagy processes appears improbable. To validate this, we evaluated the protein expression levels of large and small subunit proteins in young and old SNpc. In contrast to the findings in NMGs, SNpc did not exhibit significant differences, potentially indicating ribosomal function within NMGs themselves. Nevertheless, our techniques do not allow us to confirm ribosomal activity within post‐mortem brain tissue, thereby invalidating this hypothesis. Nonetheless, our findings encourage further investigation in this direction.

## 4 Limitations

Although the current study led to novel insights into the aging process of the human SNpc, we also must state that our presented data and results have several limitations, in particular, the low number of individuals included in each cohort, which limits the informative value of our results. Unfortunately, the availability of fresh frozen human brain tissue is scarce, especially for young age groups.

Further, our data is sex‐biased since only male individuals were assessed, due to a lack of available tissue of young female individuals. Therefore, we cannot comment on potential sex‐specific differences during the aging process in the human SNpc, although in general, differences between healthy aging in male and female individuals are frequently reported. These differences are mainly associated with sex‐chromosomal‐linked mechanisms, as previously described.^[^ ^85^ ^]^ Focusing on the brain, studies further reported sex‐specific differences in the degree of atrophy for different brain regions^[^ ^86^, ^87^ ^]^ and a generally higher resilience of females in brain aging.^[^ ^88^ ^]^ With a special focus on PD, it should also be noted that men experience an earlier disease onset and have 1.5x greater risk of developing PD, possibly due to hormonal differences. Despite that, women suffer from higher mortality rates and faster disease progression.^[^ ^89^ ^]^

A last limitation we need to highlight is the limited options to validate proteomics‐derived results, since sample specimens did not allow for the collection of large amounts of tissue. Nevertheless, our findings are in line with the current literature and corroborate recent results published in a prior study^[^ ^21^ ^]^ containing a larger cohort of young and aged individuals, in which we solely isolated SNpc tissue.

## 5 Conclusion

Our spatially‐based proteomics approach corroborated our recent findings that the aging SNpc is characterized by increased inflammation, especially in close proximity to NMGs, which may act as a “hub” of neuroinflammation. Our applied workflow further enabled us to generate information on age‐related alterations in SNpc tissue and NMGs, potentially leading to novel insights into mechanisms connecting healthy aging and neurodegeneration, such as a decreased abundance of dopaminergic marker proteins in aged individuals, potential alterations in stress granule assembly, and age‐related alterations in mitochondrial proteins. All of these effects are only observable based on the localization‐dependent approach we applied and would not have been detectable in bulk tissue studies, further encouraging studies in the research field of spatial proteomics. Our findings further underline pathomechanisms often characterized as being hallmarks of Parkinson's disease to be already observable in the physiological aging process, stressing the need for further research in understanding the aging process in the human brain, since aging is still the most important risk factor for numerous neurodegenerative diseases.

## 6 Experimental Section

### Study Cohort

Flash frozen, human post mortem Substantia nigra pars compacta (SNpc) tissue slices of 5 µm thickness were provided on 1.0 PEN‐membrane glass slides (Carl Zeiss Microscopy GmbH, Göttingen, Germany) by the Navarrabiomed Biobank (Pamplona, Spain). All tissue donors gave informed consent to use their tissue post mortem for research purposes. The use of human brain tissue was approved by the ethics committee of the RuhrUniversity Bochum, Germany (file number 4760‐13), according to German regulations and guidelines.

Tissue slices of eight control (CTRL) patients, who were identified as controls based on a combination of clinical and pathological measures by the Navarrabiomed Biobank, were used. These measures included clinical diagnosis, macroscopic evaluation of post‐mortem brain, as well as microscopic evaluation based on histological and immunohistological stainings. The latter confirmed no aggregates of tau, β‐amyloid, α‐synuclein, prion protein, ubiquitin, or TDP43 to be present in the substantia nigra. Additional information can be found in **Table** **3**.

**Table 3:** Information on post‐mortem brain tissue of young and aged donors. PMI: post‐mortem interval, SD: standard deviation.

Group	Sex	Age [in years]	Ø Age ± SD	PMI [h]	Ø PMI [h] ± SD
Young (*n* = 4)	Male	26	27.8 ± 1.3	6:12	6:18 ± 0:30
	Male	28		6:00	
	Male	28		6:00	
	Male	29		7:00	
Old (*n* = 4)	Male	62	76 ± 12.2	8:00	8:00 ± 2:57
	Male	72		9:00	
	Male	79		11:00	
	Male	91		4:00	

### Laser Microdissection and Sample Preparation

The SNpc tissue slices were stored at −80 °C until further use. The isolation of NMGs and SNpc_._ tissue was performed as previously reported.^[^ ^22^, ^23^, ^24^ ^]^ In brief, NMGs covering a tissue area of 500,000 µm^2^ and surrounding SNpc tissue devoid of NMGs (SN_Surr._), covering a tissue area of 1,000,000 µm^2^, were isolated and collected in water‐filled caps of non‐adhesive microtubes (MicroTube 500, Carl Zeiss Microscopy GmbH) using a PALM Micro Beam (P.A.L.M.‐System, Carl Zeiss Microscopy GmbH). Sample preparation was performed as reported in ref. [22]. In brief, samples were lysed in formic acid (FA) and sonicated. Samples were subsequently dried in a vacuum‐centrifuge (Concentrator plus, Eppendorf AG, Hamburg, Germany), and refilled with ammonium bicarbonate, reduced with dithiothreitol, and alkylated with iodoacetamide. Digestion was carried out using Trypsin, as explained in ref. [22] at 37 °C overnight. The digestion was stopped by adding trifluoroacetic acid (TFA), samples were vacuum‐dried again, and peptides were stored in 20 µL of 0.1% TFA at −80 °C.

### Mass Spectrometric Analysis

Liquid chromatography (LC) and mass spectrometry (MS) were carried out as described.^[^ ^22^, ^23^, ^24^ ^]^ Briefly, the LC‐MS/MS measurements were performed on an Ultimate 3000 RSLC nano LC system (ThermoFisher Scientific, Bremen, Germany) coupled to an Orbitrap Fusion Lumos Tribrid mass spectrometer (ThermoFisher Scientific, Bremen, Germany). Separation of peptides was performed at a flow rate of 400 nL min^−1^ with a linear gradient starting with 95% solution A (0.1% FA) and 5% solution B (84% acetonitrile, 0.1% FA), up to 30% B at 105 min. The nano LC‐system was directly coupled to the electrospray ionization source (ThermoFisher Scientific, Bremen, Germany) of the mass spectrometer. The mass spectrometer operated within a scan range from 350 to 1400 *m*/*z* with a resolution of 120,000 and a maximum injection time of 80 ms. In a fixed cycle time of 2 s, all precursor ions with an intensity above 1 × 10^4^ were selected for fragmentation at a fixed collision energy of 28% (HCD). Precursor ions selected for fragmentation were maintained on a dynamic exclusion list for 30 s. Fragment ion scans were performed at a resolution of 30,000 with a maximum injection time of 80 ms. ″The mass spectrometry proteomics data have been deposited to the ProteomeXchange Consortium (https://www.proteomexchange.org/) via the PRIDE partner repository^[^ ^90^ ^]^ with the dataset identifier PXD061756.

### Data Analysis

Data obtained by MS were analysed using MaxQuant (version: 1.6.10.43), with label‐free quantification (LFQ) and intensity‐based absolute quantification (iBAQ) activated. Furthermore, trypsin was selected as the protease, with a maximum of two missed cleavages allowed. The spectra were compared against a MaxQuant contaminant database and a homo sapiens reference proteome (as of January 2021) obtained from Uniprot.^[^ ^91^ ^]^ A false discovery rate (FDR) of 1% was chosen, which was determined using a reverse‐decoy database obtained from MaxQuant. Oxidation of methionine, carbamidomethylation of the N‐terminus, and deamidation of asparagine and glutamine were chosen as the dynamic modifications, while carbamidomethylation of cysteine was used as a fixed modification. The data sets obtained by MaxQuant were subsequently analysed with Perseus by first filtering out all decoys and contaminations and transforming the LFQ values logarithmically (log2(x)). For further analysis, only proteins were selected that could be identified in 75% of all samples in an age group. If values were missing, they were added by imputation using a normal distribution. A two‐tailed unpaired Student's t‐test was used to determine the significantly different proteins in both age groups. Proteins with a *p*‐value below 0.05 were considered significant, since, due to the low sample size in the cohorts, a correction of resulting *p*‐values was not feasible. Fold changes were calculated by dividing the averaged label‐free quantified values of the respective groups. Significantly differentially expressed proteins were analysed utilizing Gene Ontology association provided by Uniprot. Additionally, gene ontology term enrichment (GO‐Term) analysis on the basis of cellular compartments was performed utilizing the Database for Annotation, Visualization, and Integrated Discovery (DAVID).^[^ ^92^, ^93^ ^]^ Only GO‐Terms with a *p*‐value < 0.05 were considered for interpretation of data. Further absolute protein concentrations were calculated utilizing iBAQ values. For that, iBAQ values were normalized by calculating the sum of values for each sample separately. Single iBAQ values for each protein were subsequently divided by their respective sum. Normalized iBAQ values were additionally utilized to assess trends of abundance between young and aged individuals by calculating a fold change. Graphics were produced using R^[^ ^94^ ^]^ and the ggplot2 package.^[^ ^95^ ^]^

### Immunohistochemistry

Tissue slices on PEN membrane slides from a 29‐year‐old and a 72‐year‐old male donor were used for immunohistochemical staining. The tissue slides were first air‐dried for 10 min and then fixed using acetone. Afterward, the tissue sections were washed three times with 1xPBS. For blocking, the sections were incubated with 5% bovine serum albumin in PBS containing 0.3 Triton X‐100 for 1 h at room temperature. Subsequently, primary antibody was added in a solution containing 1% BSA in PBS with 0.3% Triton X‐100, and samples were incubated at 4 °C overnight (**Table** **4**). After 3 washing steps in 1xPBS for 5 min each, incubation with secondary antibodies followed for 1 h in the dark at room temperature (Table 4). In this step, 4′,6‐Diamidino‐2‐phenylindoldihydrochlorid (5 µg µL^−1^; Merck (Sigma–Aldrich), Darmstadt, Germany) was added in a dilution of 1:1.500. After another three washing steps, samples were covered with mounting medium (Fluoromount Aqueous Mounting Medium, Merck (Sigma–Aldrich), Darmstadt, Germany) and sealed with coverslips. Afterward, the samples were stored at 4 °C in the dark. The stained samples were afterward imaged using an Olympus BX61VS slide scanner (Olympus Soft Imaging Solutions).

**Table 4:** Utilized antibodies and corresponding dilutions.

Primary antibody	Catalogue/Lot #	Dilution	Secondary antibodies	Dilution
anti‐SERPINA1 (Alpha‐1‐antitrypsin)	PA5‐88574/ XD3568895A	1:50	Goat anti‐Rabbit IgG Alexa Fluor 555	1:500
anti‐Haptoglobin	PA5‐102426/ XD3568634	1:50	Goat anti‐Rabbit IgG Alexa Fluor 555	1:500

### Ethical Statement

Flash frozen, human post mortem Substantia nigra pars compacta (SNpc) tissue slices of 5 µm thickness were provided on 1.0 PEN‐membrane glass slides (Carl Zeiss Microscopy GmbH, Göttingen, Germany) by the Navarrabiomed Biobank (Pamplona, Spain). The use of human brain tissue was approved by the ethics committee of the Ruhr‐University Bochum, Germany (file number 4760‐13), according to German regulations and guidelines.

## Conflict of Interest

The authors declare no conflict of interest.

## Author Contributions

B.E. and M.H.contributed equally to this work. M.E., K.M. did provision of infrastructure. B.E., M.H., P.R., M.G., and K.M. did conceptualization. B.E., M.H., and K.M. did project administration. I.G.A. did collection and preparation of brain specimens.M.H. and M.L. did laser microdissection. M.L. did tryptic digestion. B.E., M.H., and M.L. did mass spectrometric measurements. B.E., M.H., M.L., K.S., B.K., and R.G. did data analysis. B.E. andM.H. did data curation. B.E., M.H., M.L., K.S., and R.G. did creation of tables and figures. B.E. and M.H. wrote the original draft. All authors reviewed the manuscript.

## Supporting information



Supporting Information (Figure S1‐S3)

Supplementary Material S1

Supplementary Material S2

Supplementary Material S3

Supplementary Material S4

Supplementary Material S5

Supplementary Material S6

Supplementary Material S7

Supplementary Material S8

Supplementary Material S9

## Data Availability

The mass spectrometry proteomics data have been deposited to the ProteomeXchange Consortium via the PRIDE partner repository with the dataset identifier PXD061756.
