# Supplementary material for: A Spatially Resolved View on the Aging Substantia nigra: An Exploratory Proteomic Study
Source: Adv Biol (Weinh). 2025 Sep 18;9(12):e00358. doi: 10.1002/adbi.202500358 (PMC12712771; doi:10.1002/adbi.202500358)
Supplement: Supplementary file 1 — Supporting Information (Figure S1‐S3) [file ADBI-9-e00358-s010.pdf]

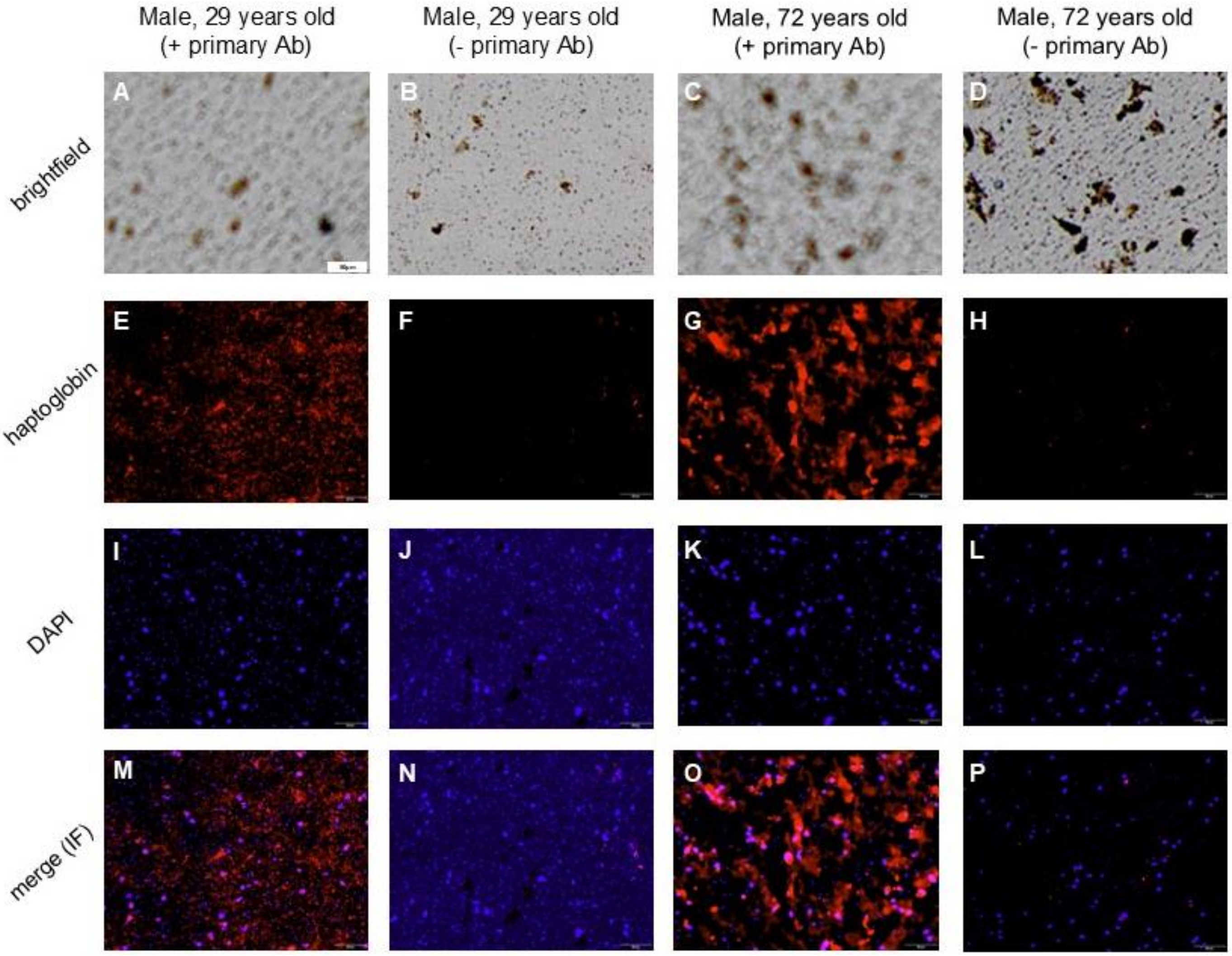


Supplementary figure S1: Immunohistochemical staining of haptoglobin (in red E-H) of substantia nigra tissue section of a healthy young 29 year old male (A, B, E, F, I, J, M, N) and an old healthy 72 year old male (C, D, G, H, K, L, O, P). Counterstaining with DAPI is visualized in I-L and a brightfield image is displayed in A-D. Overlap of all immunofluorescence (IF) channels (merge) is shown in M-P. Scale bar (A): 50µm.

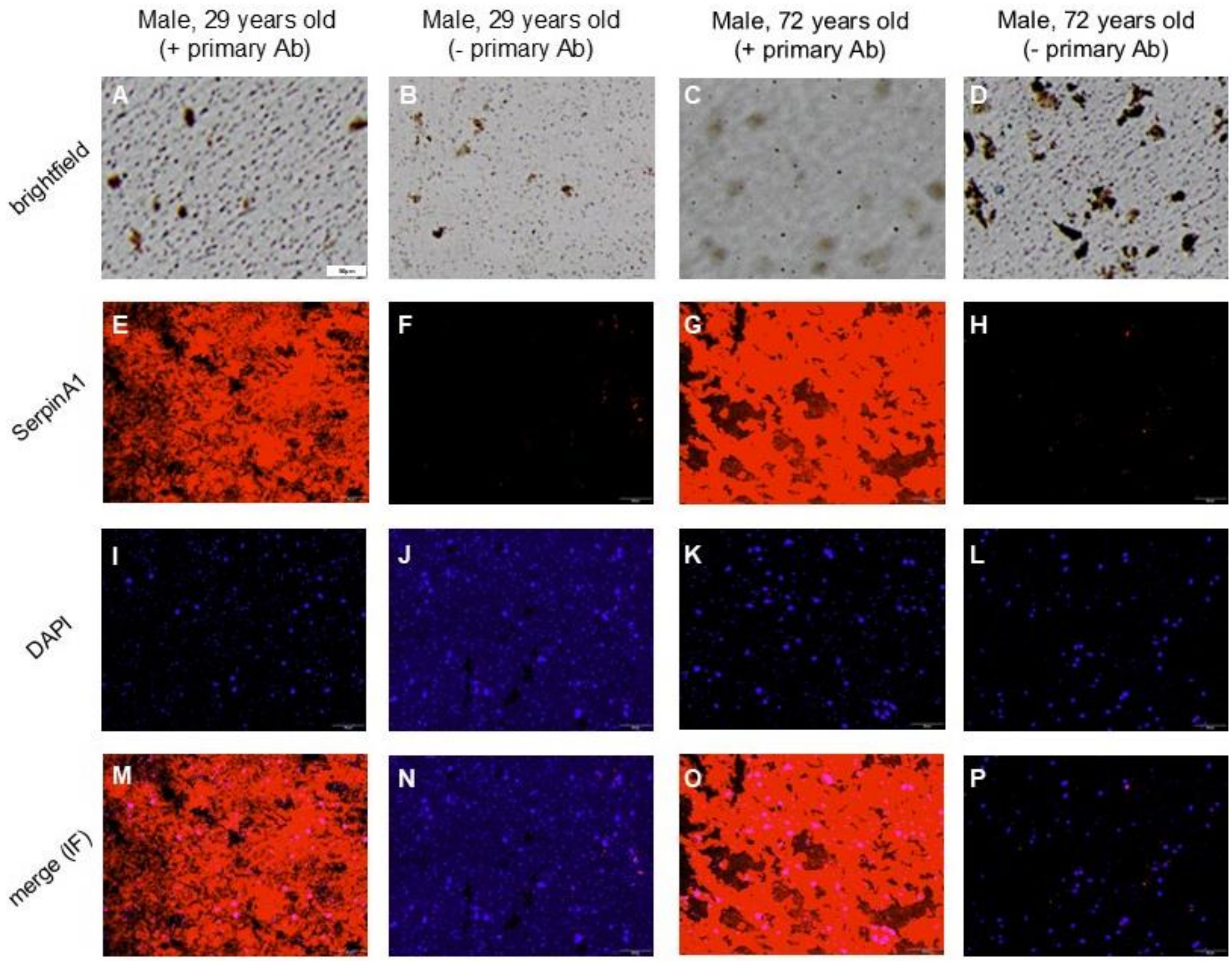


Supplementary figure S2: Immunohistochemical staining of SerpinA1 (in red E-H) of substantia nigra tissue section of a healthy young 29 year old male (A, B, E, F, I, J, M, N) and an old healthy 72 year old male (C, D, G, H, K, L, O, P). Counterstaining with DAPI is visualized in I-L and a brightfield image is displayed in A-D. Overlap of all immunofluorescence (IF) channels (merge) is shown in M-P. Scale bar (A): 50µm.

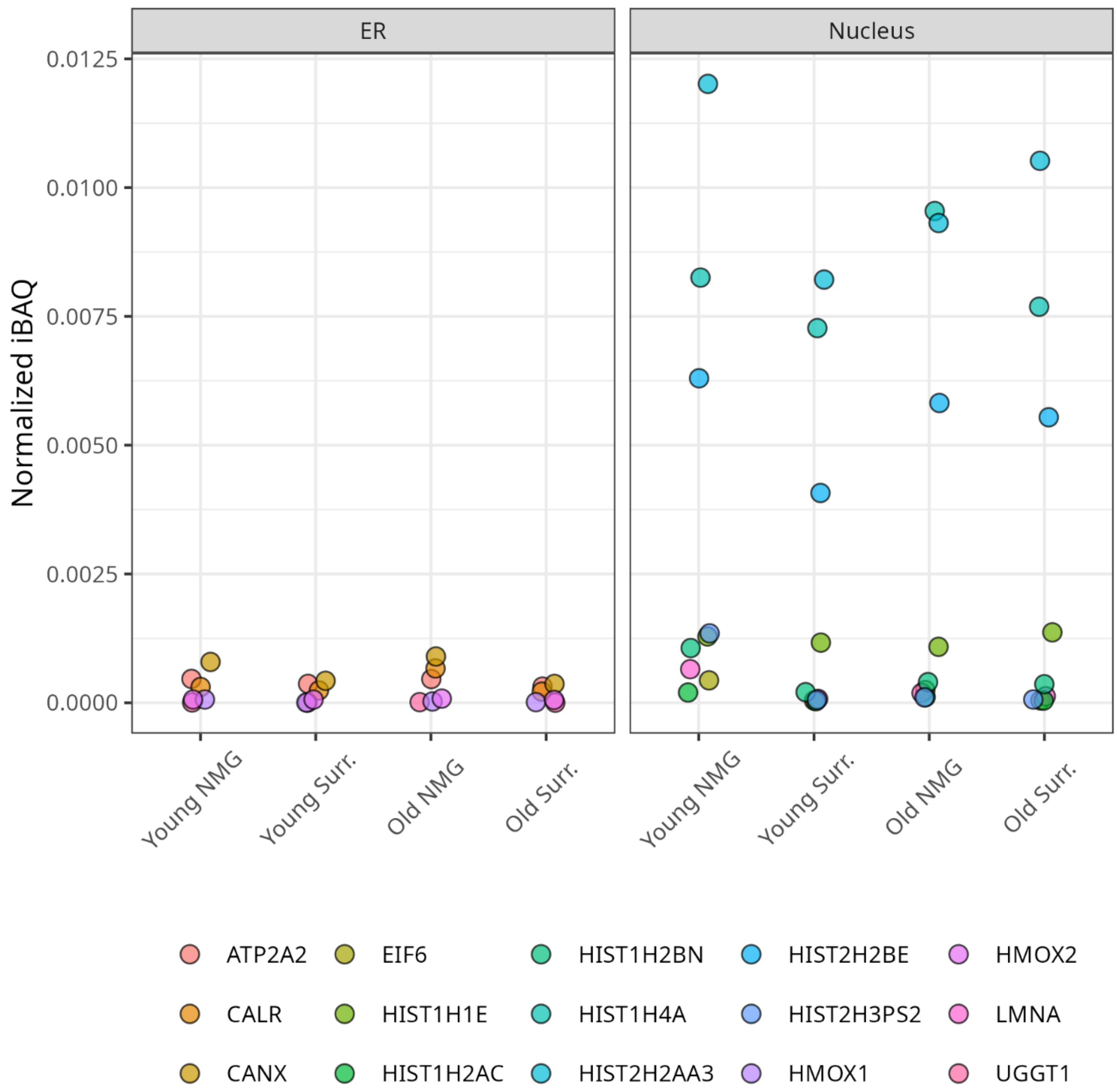


Supplementary figure S3: Distribution of abundances (based on normalized iBAQ values) of protein markers for the endoplasmic reticulum (ER) and the nucleus in young and old neuromelanin granule (NMG) samples and surrounding (Surr) substantia nigra (SN) tissue.
