# Supplementary material for: A Spatially Resolved View on the Aging Substantia nigra: An Exploratory Proteomic Study
Source: Adv Biol (Weinh). 2025 Sep 18;9(12):e00358. doi: 10.1002/adbi.202500358 (PMC12712771; doi:10.1002/adbi.202500358)
Supplement: Supplementary file 10 — Supplementary Material S9 [file ADBI-9-e00358-s008.docx]

**Supplementary information**

Supplementary figure S1: Supplementary figure S1: Immunohistochemical staining of haptoglobin (in red E-H) of substantia nigra tissue section of a healthy young 29 year old male (A, B, E, F, I, J, M, N) and an old healthy 72 year old male (C, D, G, H, K, L, O, P). Counterstaining with DAPI is visualized in I-L and a brightfield image is displayed in A-D. Overlap of all immunofluorescence (IF) channels (merge) is shown in M-P. Scale bar (A): 50µm.

Supplementary figure S2: Immunohistochemical staining of SerpinA1 (in red E-H) of substantia nigra tissue section of a healthy young 29 year old male (A, B, E, F, I, J, M, N) and an old healthy 72 year old male (C, D, G, H, K, L, O, P). Counterstaining with DAPI is visualized in I-L and a brightfield image is displayed in A-D. Overlap of all immunofluorescence (IF) channels (merge) is shown in M-P. Scale bar (A): 50µm.

Supplementary figure S3: Distribution of abundances (based on normalized iBAQ values) of protein markers for the endoplasmic reticulum (ER) and the nucleus in young and old neuromelanin granule (NMG) samples and surrounding (Surr) substantia nigra (SNpc) tissue.

Supplementary table S1: Ranked proteins (indicated by name) based on normalized iBAQ values for young and old Substantia nigra pars compacta (SNpc) tissue and neuromelanin granules (NMG)

Supplementary table S2: SNpc higher young: Proteins (ordered by gene ontology based on biological process) identified as being higher abundant in young substantia nigra pars compacta (SNpc tissue) compared to old SNpc tissue. Information on Protein ID (uniprot accession), protein name and corresponding gene name are given. Enrichment was verified by student's t-test, whereby a p-value < 0.05 was considered statistically significant. Degree of enrichment is indicated by fold change.

SNpc higher old: Proteins (ordered by gene ontology based on biological process) identified as being higher abundant in old substantia nigra pars compacta (SNpc) tissue compared to young SNpc tissue. Information on Protein ID (uniprot accession), protein name and corresponding gene name are given. Enrichment was verified by student's t-test, whereby a p-value < 0.05 was considered statistically significant. Degree of enrichment is indicated by fold change.

NMG higher young: Proteins (ordered by gene ontology based on biological process) identified as being higher abundant in young neuromelanin granules (NMGs) compared to old NMGs. Information on Protein ID (uniprot accession), protein name and corresponding gene name are given. Enrichment was verified by student's t-test, whereby a p-value < 0.05 was considered statistically significant. Degree of enrichment is indicated by fold change. NMG higher old: Proteins (ordered by gene ontology based on biological process) identified as being higher abundant in old neuromelanin granules (NMGs) compared to young NMGs. Information on Protein ID (uniprot accession), protein name and corresponding gene name are given. Enrichment was verified by student's t-test, whereby a p-value < 0.05 was considered statistically significant. Degree of enrichment is indicated by fold change.

Supplementary table 3: young SNpc: Gene ontology (GO) term enrichemnt analysis based on cellular compartments of proteins being of higher abundance in young substantia nigra pars compacta (SNpc) tissue compared to old SNpc tissue. Information on GO Term, number of and associated proteins (count, genes), percentage of term coverage (%), statistical evaluation (p-value, Bonferroni, Benjamini, FDR) and enrichment (fold enrichment) are given Old SNpc: Gene ontology (GO) term enrichment analysis based on cellular compartments of proteins being of higher abundance in old substantia nigra pars compacta (SNpc) tissue compared to young SNpc tissue. Information on GO Term, number of and associated proteins (count, genes), percentage of term coverage (%), statistical evaluation (p-value, Bonferroni, Benjamini, FDR) and enrichment (fold enrichment) are given. Young NMG: Gene ontology (GO) term enrichemnt analysis based on cellular compartments of proteins being of higher abundance in young neuromelanin granules (NMGs) compared to old NMGs. Information on GO Term, number of and associated proteins (count, genes), percentage of term coverage (%), statistical evaluation (p-value, Bonferroni, Benjamini, FDR) and enrichment (fold enrichment) are given. Old NMG: Supplementary table 3: Gene ontology (GO) term enrichemnt analysis based on cellular compartments of proteins being of higher abundance in old neuromelanin granules (NMGs) compared to young NMGs. Information on GO Term, number of and associated proteins (count, genes), percentage of term coverage (%), statistical evaluation (p-value, Bonferroni, Benjamini, FDR) and enrichment (fold enrichment) are given

Supplementary table S4: normalized iBAQ values of young and old substantia nigra pars compacta (SNpc) and neuromelanin granules (NMGs) of proteins associated with the clathrin-mediated transport of vesicles. Proteins are indicated by gene name and sorted into differnt functional categories.

Supplementary table S5: Mitochondrial proteins NMGs: Identified mitochondrial proteins (according to Top100 mitochondrial proteins annotated by MitoCarta 3.0) and their respective normalized iBAQ values in young and old neuromelanin granules (NMG) and calculated fold change (FC) between iBAQ values.

Mitochondrial proteins SNpc: Identified mitochondrial proteins (according to Top100 mitochondrial proteins annotated by MitoCarta 3.0) and their respective normalized iBAQ values in young and old substantia nigra (SNpc) and calculated fold change (FC) between iBAQ values. Overlap comparison: Overlap comparison of mitochondrial proteins present in all sample types, including ratios of iBAQ values between young/old neuromelanin granules (NMG) and young/old substantia nigra (SNpc) tissue. Overlapping proteins: Overlap of mitochondrial proteins present in all sample types, including ratios of iBAQ values between young/old neuromelanin granules (NMG) and young/old substantia nigra (SNpc) tissue.

Supplementary table S6: Stress granule-associated proteins (indicated by gene name) and respective calculated normalized iBAQ values for each protein in young and old neuromelanin granules (NMG). Additionally a ratio was calculated.

Supplementary table S7: Normalized iBAQ values of proteins associated with the large and small ribosomal subunit in young and old Substantia nigra pars compacta (SNpc) and neuromelanin granules (NMG)

Supplementary table S8: Normalized iBAQ values of proteins associated withthe nucleus and endoplasmic reticulum (ER) in young and old Substantia nigra pars compacta (SNpc) and neuromelanin granules (NMG)
